# Associations between the intake of single and multiple dietary vitamins and depression risk among populations with chronic kidney disease

**DOI:** 10.3389/fnut.2025.1492829

**Published:** 2025-02-04

**Authors:** Chunli Yu, Kun Liu, Weiguo Yao, Dingzhong Tang

**Affiliations:** ^1^Department of Nephrology, Jinshan Branch of Shanghai Sixth People's Hospital, Shanghai, China; ^2^Department of Neurology, Jinshan Branch of Shanghai Sixth People's Hospital, Shanghai, China

**Keywords:** dietary vitamin intake, depression, risk, chronic kidney disease, multiple, single

## Abstract

**Background:**

The effects of multivitamin exposure on depression among patients with chronic kidney disease (CKD) have not been thoroughly explored. This study aimed to explore the effects of individual vitamin intakes and the joint effect of the intake of multiple vitamins (including vitamins A, B_1_, B_2_, B_6_, B_12_, C, D, E, and K) on depression risk in participants with CKD.

**Methods:**

A total of 3,123 participants with CKD (weighted *n* = 25,186,480) from the National Health and Nutrition Examination Survey database from 2007 to 2014 were included. Weighted multivariate logistic regression models were utilized to analyze the associations of individual dietary vitamin intakes with depression risk. Additionally, Bayesian kernel machine regression (BKMR) and weighted quantile sum (WQS) regression were performed to evaluate the joint effect of the intake of the nine vitamins on depression risk.

**Results:**

The overall prevalence of depression was approximately 11.3% in the study participants. In the fully adjusted model, high intakes of vitamin A (OR: 0.54, 95% CI: 0.40–0.74), vitamin B_1_ (OR: 0.67, 95% CI: 0.48–0.95), vitamin B_6_ (OR: 0.70, 95% CI: 0.49–0.99), vitamin D (OR: 0.67. 95% CI: 0.48–0.94), and vitamin K (OR: 0.61, 95% CI: 0.44–0.85) were associated with a reduced likelihood of depression. BKMR and WQS regression showed that the joint effect of the intake of the nine dietary vitamins had a significant negative effect on depression, with vitamin A intake being the largest contributor in the two models. Lastly, WQS regression reflected the total mixed exposure effect of the nine vitamins (OR: 0.82, 95% CI: 0.69–0.99).

**Conclusion:**

High intakes of vitamins A, B_1_, B_6_, D, and K are associated with low depression risk in patients with CKD. Furthermore, co-exposure to the nine dietary vitamins is a crucial factor contributing to low depression risk in this population.

## Introduction

Chronic kidney disease (CKD) is a prevalent and progressive condition characterized by the gradual loss of kidney function, affecting millions of individuals worldwide (1). As the disease progresses, patients often experience a myriad of complications, such as cardiovascular disease, bone disorders, and an increased susceptibility to infections (2–4). Among these complications, the mental health of patients with CKD is of notable concern, with depression being a common but under-recognized and undertreated condition in this population. Depression not only deteriorates the quality of life but also contributes to relatively poorer clinical outcomes, including higher CKD morbidity and mortality (5, 6).

Depression in patients with CKD has a complex and multifactorial pathophysiology involving biochemical, psychosocial, and lifestyle factors (7, 8). An area of growing interest is the potential role of nutritional deficiencies, particularly vitamin deficiencies, in depression development and progression among patients with CKD. Vitamins are essential micronutrients that are crucially involved in various physiological processes, including neurotransmitter synthesis, immune function, and oxidative stress regulation (9–11). Deficiencies in certain vitamins, such as vitamin D, the B vitamins, and antioxidants such as vitamin C, have been implicated in depression pathogenesis (9, 12). Given the altered metabolism and usual dietary restrictions in patients with CKD, vitamin deficiencies are particularly widespread in this group (13, 14). Furthermore, CKD-related inflammation and oxidative stress may be exacerbated by these deficiencies, thereby escalating depression risk (15). Although a substantial body of research is available on the associations between individual vitamin intake and depression, the effects of multivitamin exposure on depression among patients with CKD have not been comprehensively explored.

Generally, various populations are typically exposed to multiple dietary vitamins simultaneously, which may produce synergistic or antagonistic effects (16). Therefore, determining the effects of the intake of multiple vitamins on depression in patients with CKD is critical. In contrast to previous studies that have assessed the relationship between individual vitamin exposure and depression, our study aims to investigate the associations of single and multiple dietary vitamin intakes with depression risk in patients with CKD. According to earlier literature reports (17), the vitamins included in this study were vitamins A, B_1_, B_2_, B_6_, B_12_, C, D, E, and K. This study examines a comprehensive range of vitamins to provide a deeper understanding of the influence of nutritional intake on the mental health of this vulnerable population. The findings from this investigation may have significant implications for developing targeted dietary interventions and supplementation strategies to improve mental health outcomes in patients with CKD.

## Materials and methods

### Study design and population

**Figure 1:**
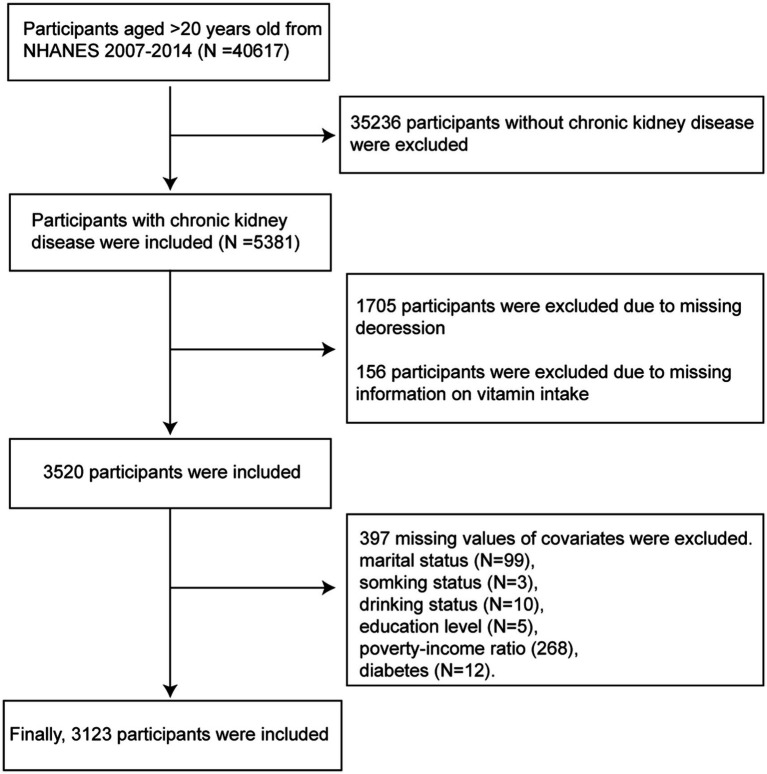
The flowchart of participants included in the study.

This cross-sectional study utilized the 2007–2014 data from the National Health and Nutrition Examination Survey (NHANES), a program designed to assess the health and nutritional status of adults in the United States. NHANES employs a complex, multistage probability sampling design to ensure that the survey sample is representative of the civilian non-institutionalized U.S. population. The study population included participants with a diagnosis of CKD, which was established based on an estimated glomerular filtration rate of <60 mL/min/1.73 m^2^ or albuminuria (urinary albumin-to-creatinine ratio ≥ 30 mg/g) for 3 months or more. Participants who were ≥ 20 years of age and had complete data on dietary vitamin intake, depression status, and other covariates were included. The detailed participant inclusion process of our study is illustrated in Figure 1.

The NHANES protocol was approved by the National Center for Health Statistics Research Ethics Review Board, and all included participants provided written informed consent. The survey adhered to the ethical principles of the Declaration of Helsinki. Furthermore, no additional ethical approval was required for this study due to its retrospective nature.

### Dietary vitamin assessment

The vitamins examined in this study included vitamins A, B_1_, B_2_, B_6_, B_12_, C, D, E, and K. Vitamin intake was evaluated through dietary intake and dietary supplement intake. The total daily intake of vitamins was determined by summing up the data obtained from a 24-h dietary recall interview and the daily vitamin intake from the dietary supplements of the participants. The total daily vitamin intake was estimated as the sum of the daily intake of the nine vitamins.

### Assessment of depression

Depression status was assessed using the Patient Health Questionnaire-9 (PHQ-9), a validated self-report tool that measures the severity of depressive symptoms over the past 2 weeks. The PHQ-9 scores range from 0 to 27, with higher scores indicating more severe depressive symptoms. In this study, participants with a PHQ-9 score of ≥10 were classified as having depression.

### Covariates

This analysis considered several potential confounding variables, including demographic characteristics (age, sex, ethnicity, marital status, poverty-income ratio, and education level), lifestyle factors (smoking and drinking status), and comorbidities (hypertension, diabetes, and hyperlipidemia). Data on these variables were collected through standardized interviews and physical examinations conducted during the NHANES. Detailed information on the analyzed covariates can be obtained.^1^

### Statistical analysis

All statistical analyses were performed after considering the survey design and sample weights to ensure that the results were representative of the US population. Descriptive statistics were calculated for all variables. Categorical variables were expressed as percentages and compared using the *X*^2^ test. Furthermore, continuous variables were presented as mean ± standard deviation or median, and group comparisons were conducted through the *t*-test or Wilcoxon test.

Dose–response analysis was performed using weighted restricted cubic spline functions with three knots located at the 5, 50, and 95th percentiles of individual dietary vitamin intakes. The Wald chi-square test with adjustments for age, sex, ethnicity, marital status, poverty-income ratio, education level, smoking status, drinking status, diabetes, hyperlipidemia, and hypertension was employed to assess the potential linear associations between the intakes of individual dietary vitamins and mortality risks. Additionally, weighted logistic regression models were established to explore the associations between the intakes of individual vitamins and depression risk. Model 1 was not adjusted for the confounding variables, whereas model 2 was adjusted for age, sex, ethnicity, marital status, poverty-income ratio, education level, smoking status, drinking status, diabetes, hyperlipidemia, and hypertension. The variance inflation factor (VIF) was calculated to evaluate multicollinearity between variables (Supplementary Table S1). Based on previous studies, variables with VIF > 5 were considered collinear variables (18) and were deleted in the final model. The VIFs of the variables included in the final model were found in Supplementary Table S1.

Finally, we used two mixed analysis methods—Bayesian kernel machine regression (BKMR) and weighted quantile sum (WQS) regression—to evaluate the joint effect of the intake of nine dietary vitamins on depression risk. The BKMR model integrates Bayesian and statistical learning methodologies to assess the nonlinear and/or interactive effects of the associations between exposures and outcomes. The correlation coefficients among the intakes of the nine dietary vitamins were computed utilizing Pearson correlation analysis. Subsequently, the intakes of the nine dietary vitamins were categorized according to their correlation coefficient plots. Group posterior inclusion probability (GroupPIP) and conditional posterior inclusion probability (CondPIP) were then used as metrics to quantify the likelihood of each group and dietary vitamin intake being incorporated into the model, thereby elucidating their respective contributions to the overall effect. In the present study, BKMR was performed utilizing the R package “bkmr” to determine the joint effect of the intake of the nine dietary vitamins on depression risk. Similarly, the WQS regression model was implemented using the R package “WQS” to estimate the WQS index, which evaluates the combined effect of co-exposure to the nine dietary vitamins, along with the contribution of each dietary vitamin exposure (19). The WQS index was calculated based on the weighted sum of the intake of the nine dietary vitamins. The weight assigned to each dietary vitamin in the WQS index reflects its specific contribution to the overall exposure effect.

All statistical analyses were performed using R 4.4.0 software, and a *p*-value of <0.05 denoted statistically significant differences.

## Results

### Baseline characteristics of the study participants

A total of 3,123 participants with CKD (weighted *n* = 25,186,480) were included in this study. The participants had an average age of 62.9 ± 16.21 years, while the distribution of women and men was 51.75 and 48.25%, respectively. The baseline characteristics of the included participants are shown in Table 1. The overall prevalence of depression was approximately 11.3% (*n* = 353). Compared with the group without depression, the depression group had a younger age, a higher proportion of women, a lower education level, and higher incidences of hyperlipidemia and diabetes (*p*-values < 0.05). Additionally, the baseline characteristics of participants grouped by CKD stages are provided in Supplementary Table S2. Except for education level and vitamin K intake, all other variables exhibited significant differences among the groups (*p*-values <0.05).

**Table 1:** Baseline characteristics of the present study.

Variables	Participants, No. (%)			*p*-value
	Total *N* = 3,123	No depression	Depression	
		*N* = 2,770	*N* = 353	
Age, years				<0.001
≤65	1,484(47.52)	1,259(45.45)	225(63.74)	
>65	1,639(52.48)	1,511(54.55)	128(36.26)	
Sex				<0.001
Male	1,507(48.25)	1,385(50.00)	122(34.56)	
Female	1,616(51.75)	1,385(50.00)	231(65.44)	
Ethnicity				<0.01
White	1,604(51.36)	1,451(52.38)	153(43.34)	
Mexican	364(11.66)	311(11.23)	53(15.01)	
Black	700(22.41)	614(22.17)	86(24.36)	
Other	455(14.57)	394(14.22)	61(17.28)	
Marital status				<0.001
Married	1,559(49.92)	1,431(51.66)	128(36.26)	
Other	1,564(50.08)	1,339(48.34)	225(63.74)	
Education level				<0.001
Less than high school graduate	979(31.35)	826(29.82)	153(43.34)	
High school graduate or general equivalency diploma	765(24.50)	690(24.91)	75(21.25)	
Some college or above	1,379(44.16)	1,254(45.27)	125(35.41)	
PIR				<0.001
<1.0	707(22.64)	565(20.40)	142(40.23)	
≥1.0	2,416(77.36)	2,205(79.60)	211(59.77)	
Smoking status				<0.001
Never	1,535(49.15)	1,387(50.07)	148(41.93)	
Former	1,039(33.27)	937(33.83)	102(28.90)	
Now	549(17.58)	446(16.10)	103(29.18)	
Drinking status				<0.001
Never	532(17.03)	473(17.08)	59(16.71)	
Former	910(29.14)	775(27.98)	135(38.24)	
Now	1,681(53.83)	1,522(54.95)	159(45.04)	
Diabetes				<0.001
No	1840(58.92)	1,674(60.43)	166(47.03)	
Yes	1,283(41.08)	1,096(39.57)	187(52.97)	
Hyperlipidemia				0.03
No	562(18.00)	514(18.56)	48(13.60)	
Yes	2,561(82.00)	2,256(81.44)	305(86.40)	
Hypertension				0.34
No	859(27.51)	770(27.80)	89(25.21)	
Yes	2,264(72.49)	2000(72.20)	264(74.79)	
Vitamin Types, Median (IQR)				
Vitamin A, μg	467.00(500.5)	478.00(502.75)	355.00(446.00)	<0.001
Vitamin B1, mg	1.29(0.92)	1.31(0.91)	1.17(0.92)	<0.01
Vitamin B2, mg	1.70(1.18)	1.72(1.18)	1.61(1.10)	0.04
Vitamin B6, mg	1.59(1.21)	1.62(1.20)	1.37(1.27)	<0.001
Vitamin B12, μg	3.58(3.72)	3.64(3.77)	3.07(3.36)	<0.01
Vitamin C, mg	50.10(85.45)	51.75(86.27)	38.80(82.00)	0.33
Vitamin D, μg	3.20(4.45)	3.20(4.40)	2.70(4.60)	0.01
Vitamin E, mg	5.84(5.19)	5.92(5.22)	5.21(5.25)	0.03
Vitamin K, ug	55.00(75.80)	56.60(77.90)	43.90(52.40)	0.02
Total vitamin intake, mg	63.53 (90.02)	66.21 (90.74)	51.38 (81.48)	<0.001

PIR, poverty-income ratio; IQR, interquartile range (75th quartile minus 25th quartile).

### Associations between the intakes of individual vitamins and depression risk

**Figure 2:**
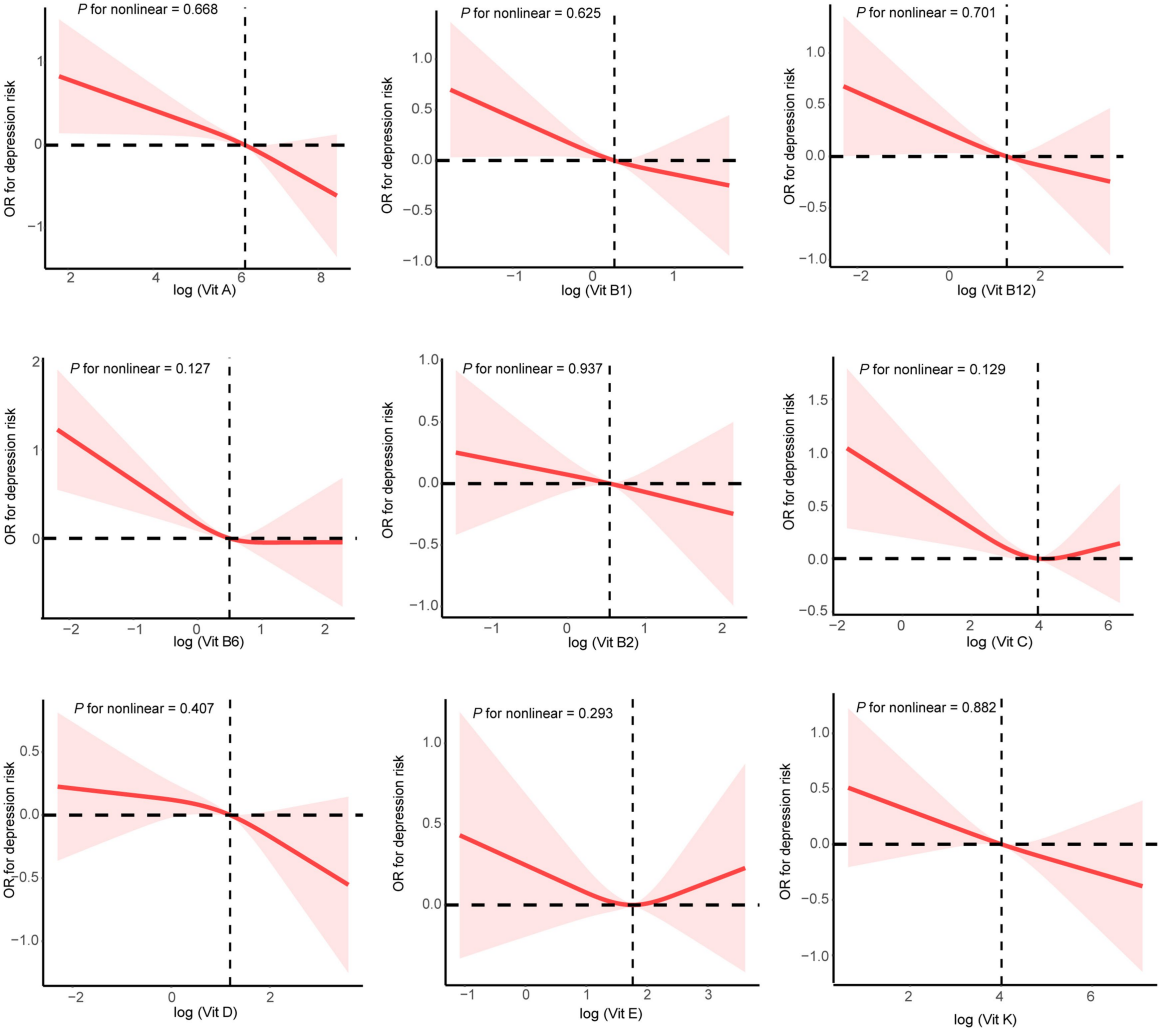
Restricted cubic spline plots for single dietary vitamin intake and depression risk. Age, sex, ethnicity, marital status, poverty-income ratio, education level, smoking status, drinking status, diabetes, hyperlipidemia, and hypertension were adjusted.

The dose–response curves between the intakes of the nine dietary vitamins and depression risk are presented in Figure 2. The intakes of the nine dietary vitamins showed a negative linear association with depression risk among the participants with CKD. Table 2 depicts the relationships between the intakes of individual dietary vitamins and depression risk. In the weighted univariate logistic regression model (model 1), high intakes of the nine dietary vitamins were associated with a lower depression risk. After adjusting for age, sex, ethnicity, marital status, poverty-income ratio, education level, smoking status, drinking status, diabetes, hyperlipidemia, and hypertension (model 2), high intakes of vitamin A (OR: 0.54, 95% CI: 0.40–0.74), vitamin B_1_ (OR: 0.67, 95% CI: 0.48–0.95), vitamin B_6_ (OR: 0.70, 95% CI: 0.49–0.99), vitamin D (OR: 0.67. 95% CI: 0.48–0.94), and vitamin K (OR: 0.61, 95% CI: 0.44–0.85) were found to be associated with a reduced likelihood of depression.

**Table 2:** Single dietary vitamin intake and depression risk.

Variables	Model 1		Model 2	
	OR 95%CI	*p*-value	OR 95%CI	*p*-value
Tertiles of vitamin A, μg				
Q1	ref		ref	
Q2	0.58(0.44,0.77)	<0.001	0.71(0.54,0.94)	0.02
Q3	0.43(0.33,0.56)	<0.001	0.54(0.40,0.74)	<0.001
Tertiles of vitamin B1, mg				
Q1	ref		ref	
Q2	0.75(0.49,1.14)	0.17	0.95(0.61,1.48)	0.83
Q3	0.56(0.40,0.77)	<0.001	0.67(0.48,0.95)	0.03
Tertiles of vitamin B2, mg				
Q1	ref		ref	
Q2	0.75(0.53,1.07)	0.11	0.85(0.57,1.26)	0.40
Q3	0.60(0.43,0.84)	0.003	0.74(0.53,1.03)	0.08
Tertiles of vitamin B6, mg				
Q1	ref		ref	
Q2	0.57(0.42,0.78)	<0.001	0.64(0.46,0.89)	0.01
Q3	0.56(0.40,0.78)	<0.001	0.70(0.49,0.99)	0.046
Tertiles of vitamin B12, μg				
Q1	ref		ref	
Q2	0.75(0.48,1.19)	0.220	0.75(0.47,1.20)	0.22
Q3	0.68(0.49,0.93)	0.018	0.79(0.56,1.12)	0.19
Tertiles of vitamin C, mg				
Q1	ref		ref	
Q2	0.57(0.39,0.83)	0.004	0.72(0.48,1.08)	0.11
Q3	0.57(0.43,0.77)	<0.001	0.79(0.57,1.11)	0.17
Tertiles of vitamin D, μg				
Q1	ref		ref	
Q2	0.64(0.44,0.94)	0.02	0.74(0.50,1.10)	0.13
Q3	0.60(0.43,0.83)	0.003	0.67(0.48,0.94)	0.02
Tertiles of vitamin E, mg				
Q1	ref		ref	
Q2	0.84(0.60,1.17)	0.30	0.96(0.67,1.37)	0.82
Q3	0.79(0.54,1.15)	0.21	1.03(0.68,1.56)	0.88
Tertiles of vitamin K, μg				
Q1	ref		ref	
Q2	0.85(0.59,1.23)	0.38	1.01(0.69,1.46)	0.97
Q3	0.48(0.34,0.67)	<0.001	0.61(0.44,0.85)	0.01

Model 1: No variables were adjusted. Model 2: Age, sex, ethnicity, marital status, poverty-income ratio, education level, smoking status, drinking status, diabetes, hyperlipidemia, and hypertension were adjusted. OR, odds ratio; CI, confidence interval.

### Association of exposure to multiple dietary vitamins and depression risk

**Figure 3:**
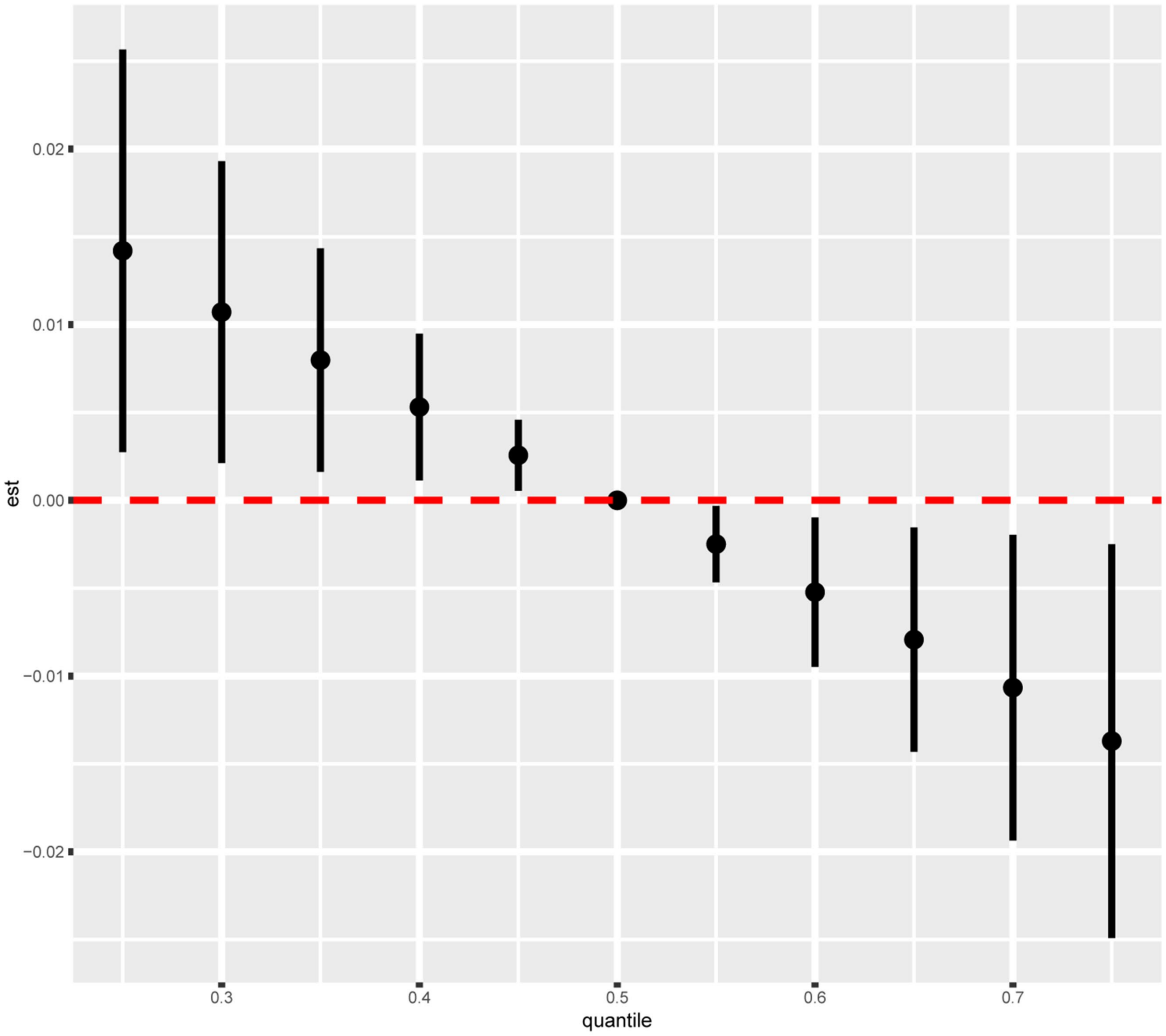
Combined effects of nine dietary vitamins mixtures and depression by BKMR analysis. Model was adjusted for age, sex, ethnicity, marital status, poverty-income ratio, education level, smoking status, drinking status, diabetes, hyperlipidemia, and hypertension.

The combined effect of the intake of the nine dietary vitamins on depression was determined using the BKMR model. As shown in the fully adjusted BKMR model in Figure 3, the overall effect of the intake of the nine dietary vitamins showed a decreasing trend in depression occurrence in participants with CKD. Furthermore, an increase in the total intake level of the nine dietary vitamins was negatively correlated with depression risk. Additionally, the correlations among the intakes of the nine dietary vitamins are illustrated in Supplementary Figure S1. In particular, vitamin B_1_ intake was significantly correlated with vitamins B_2_ intake (*r* = 0.75) and B_6_ intake (*r* = 0.66), while vitamin B_2_ intake exhibited correlations with vitamins B_6_ intake (*r* = 0.65), B_12_ intake (*r* = 0.69), and D intake (*r* = 0.60). A notable correlation was also observed between vitamin B_12_ intake and vitamins B_6_ intake (*r* = 0.61) and D intake (*r* = 0.64). Finally, a correlation was detected between vitamin E intake and K intake (*r* = 0.59). Subsequently, the nine dietary vitamins were grouped according to the correlation analysis results described above. The GroupPIP and CondPIP values of the nine dietary vitamins derived from the BKMR model are summarized in Supplementary Table S3. The GroupPIP of group 1 (vitamin A: 0.64) was higher than that of the other three groups (vitamin B_1_, B_2_, B_6_, B_12_, and D: 0.53; vitamin C: 0.29; and vitamins E and K: 0.61). Moreover, the CondPIP values of vitamin A (CondPIP = 1.0) and vitamin C (CondPIP = 1.0) were higher than those of the other dietary vitamins. Therefore, dietary vitamin A contributed the most to the BKMR model of depression risk. Furthermore, depression risk decreased with increased intakes of vitamins A, B_6_, and K and the median intake levels of all remaining dietary vitamins (Supplementary Figure S2). Single exposure-response functions for each dietary vitamin intake showed that significant associations between single vitamin intake and depression were weaker when holding other vitamin intakes at the 25th, 50th, and 75th percentiles (Supplementary Figure S3). Bivariate exposure-response functions (Supplementary Figure S4) for each dietary vitamin intake demonstrated no potential interactions among the dietary vitamins.

As shown in Table 3, WQS regression analysis revealed that co-exposure to the nine dietary vitamins was negatively associated with depression risk (OR: 0.82, 95% CI: 0.69–0.99). The estimated weights of the nine dietary vitamins in the WQS model were also calculated (Supplementary Table S4; Supplementary Figure S5). Vitamin A was found to be the dietary vitamin with the highest weight in the WQS model, followed by vitamin K.

**Table 3:** The combined effect of nine dietary vitamins on depression by WQS model.

Model	OR 95%CI	*p*-value
WQS model	0.82 (0.69, 0.99)	0.035

Model was adjusted for age, sex, ethnicity, marital status, poverty-income ratio, education level, smoking status, drinking status, diabetes, hyperlipidemia, and hypertension. WQS, weighted quantile sum; OR, odds ratio; CI, confidence interval.

## Discussion

In this study, we investigated the associations between the intake of single and multiple dietary vitamins and depression risk among participants with CKD using data from the NHANES database. Our findings showed that high intakes of certain vitamins, particularly vitamins A, B_1_, B_6_, D, and K, were associated with a low depression risk in this population. Moreover, the combined deficiency of such multiple vitamins might exacerbate depression risk, highlighting the significance of a comprehensive approach to nutritional management in patients with CKD.

A study by Liu et al. found that depression prevalence among adults with CKD in the United States was 10.97% (20), which was extremely similar to the depression prevalence of 11.3% reported in this study. This finding demonstrates that the participant sample used in this study is representative of this patient population. Additionally, this study revealed that participants with CKD as well as those with depression had lower vitamin intakes than the daily recommendation. For example, the recommended vitamin A intake for adults is 700 μg/day for women and 900 μg/day for men (21). In our study, the average daily intake of vitamin A in participants with CKD was 560.83 μg/day in women and 644.46 μg/day in men. Furthermore, participants with CKD but without depression had an average daily intake of vitamin A of 577.45 μg/day in women and 650.04 μg/day in men, with this intake being further reduced among those with CKD accompanied by depression (461.19 μg/day in women and 581.04 μg/day in men). Hence, adequate vitamin supplementation may be a vital strategy to prevent depression among patients with CKD.

Moreover, our results were consistent with previous studies that have identified a link between vitamin intake and depression in the general population and those with chronic illnesses. For instance, a high intake of vitamin A has been widely reported to correlate with low depression risk among patients with heart failure, potentially due to its role in inflammation regulation (22, 23). Correspondingly, deficiencies in the B vitamins, particularly B_1_ and B_6_, have been associated with depression. Xu et al. reported that a thiamine (vitamin B_1_) intake of <1.35 mg/day was negatively correlated with depression incidence (OR: 0.68, 95% CI: 0.53–0.89) (24). Another investigation by Ekinci et al. suggested an association of vitamin B_6_ intake with depression risk (25). The pathogenesis of neuropsychiatric diseases such as depression is related to neuroinflammation, oxidative stress, and cell apoptosis (26). Therefore, the reduction of depression by the B vitamins may be due to their inhibition of apoptosis, oxidative damage, neuroinflammation, and caspase-1-mediated inflammasome activation (27). Additionally, the study revealed that vitamin K intake was negatively and independently associated with depression incidence in American adults (OR: 0.84, 95% CI: 0.75–0.94) (28). In the case of vitamin D intake, patients with CKD not only have inadequate nutrition and sunlight exposure but also exhibit impaired synthesis and metabolism of vitamin D. Therefore, vitamin D deficiency is more pronounced in patients with CKD (29). A study by Jhee et al. showed that patients with CKD had a higher depression incidence, with vitamin D deficiency being a significant independent predictor of depression (OR: 6.15; 95% CI: 2.02–8.75). Consequently, alleviating vitamin D deficiency could help prevent depression occurrence (30). In line with the findings from these previous studies, our study demonstrated that high intakes of vitamins A, B_1_, B_6_, D, and K were associated with a lower depression risk. Moreover, our study results extend the earlier findings by demonstrating that the cumulative effect of multiple dietary vitamins intake may have a more pronounced effect on lowering depression risk among participants with CKD (OR: 0.82, 95% CI: 0.69–0.99).

Patients with CKD have a high prevalence of vitamin deficiencies, which further elevates the risk of depression in this population (31, 32). Therefore, proactive nutritional assessment and intervention are crucial in this patient group. Consistent with this notion, previous studies have shown that regularly monitoring the levels and dietary intake of vitamins, along with appropriate supplementation, may be a key strategy to mitigate depression risk and improve overall mental health outcomes (33, 34). Furthermore, considering the complex interactions between CKD, inflammation, and nutrient absorption, a personalized nutritional plan should be incorporated into a comprehensive treatment approach for patients with CKD. Thus, the present study findings could have substantial implications for the development of targeted dietary interventions and supplementation strategies to improve the mental health of patients with CKD.

One of the strengths of the current study was the use of a large, nationally representative sample from the NHANES database, which enhances the generalizability of our findings to the broader U.S. population with CKD. However, our study has several limitations that should be acknowledged. First, the cross-sectional design of this study prevented us from establishing causality between vitamin deficiencies and depression. Hence, longitudinal studies are required to determine whether alleviating these vitamin deficiencies can reduce depression risk over time. Second, dietary intake was assessed using a 24-h dietary recall interview, which may have resulted in recall bias and not accurately reflected the long-term intake of vitamins. Finally, this study adjusted for a range of potential confounders; however, residual confounding due to unmeasured factors cannot be completely ruled out. Therefore, future research should involve longitudinal studies to explore the causal relationships between vitamin intake and depression in patients with CKD. Additionally, interventional studies are necessary to evaluate the effectiveness of vitamin supplementation in preventing and treating depression in this patient population. Finally, research elucidating the mechanisms by which vitamin deficiencies contribute to depression in CKD may also provide insights into novel therapeutic targets.

## Conclusion

This cross-sectional study utilizing data from the 2007–2014 NHANES revealed a significant inverse correlation between the intakes of vitamins A, B_1_, B_6_, D, and K and depression risk in participants with CKD. Additionally, co-exposure to the nine dietary vitamins was found to be a critical factor contributing to the mental health of this specific population. Our study results emphasize the need for comprehensive nutritional assessments and interventions as part of the routine care for patients with CKD, ultimately aiding to improve their physical and mental health outcomes.

## Data Availability

Publicly available datasets were analyzed in this study. This data can be found at: https://www.cdc.gov/nchs/nhanes/.

## Footnotes

^1^ https://www.cdc.gov/nchs/nhanes/
