# Supplementary figures and images for: Associations between the intake of single and multiple dietary vitamins and depression risk among populations with chronic kidney disease
Source: Front Nutr. 2025 Feb 4;12:1492829. doi: 10.3389/fnut.2025.1492829 (PMC11832393; doi:10.3389/fnut.2025.1492829)

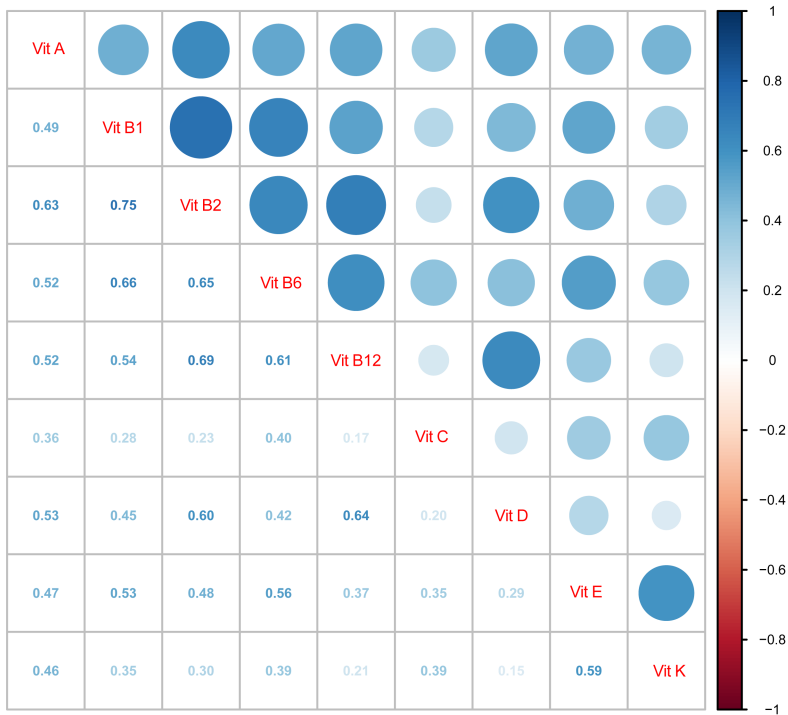

Supplement: Supplementary file 1 [file Image_1.pdf]

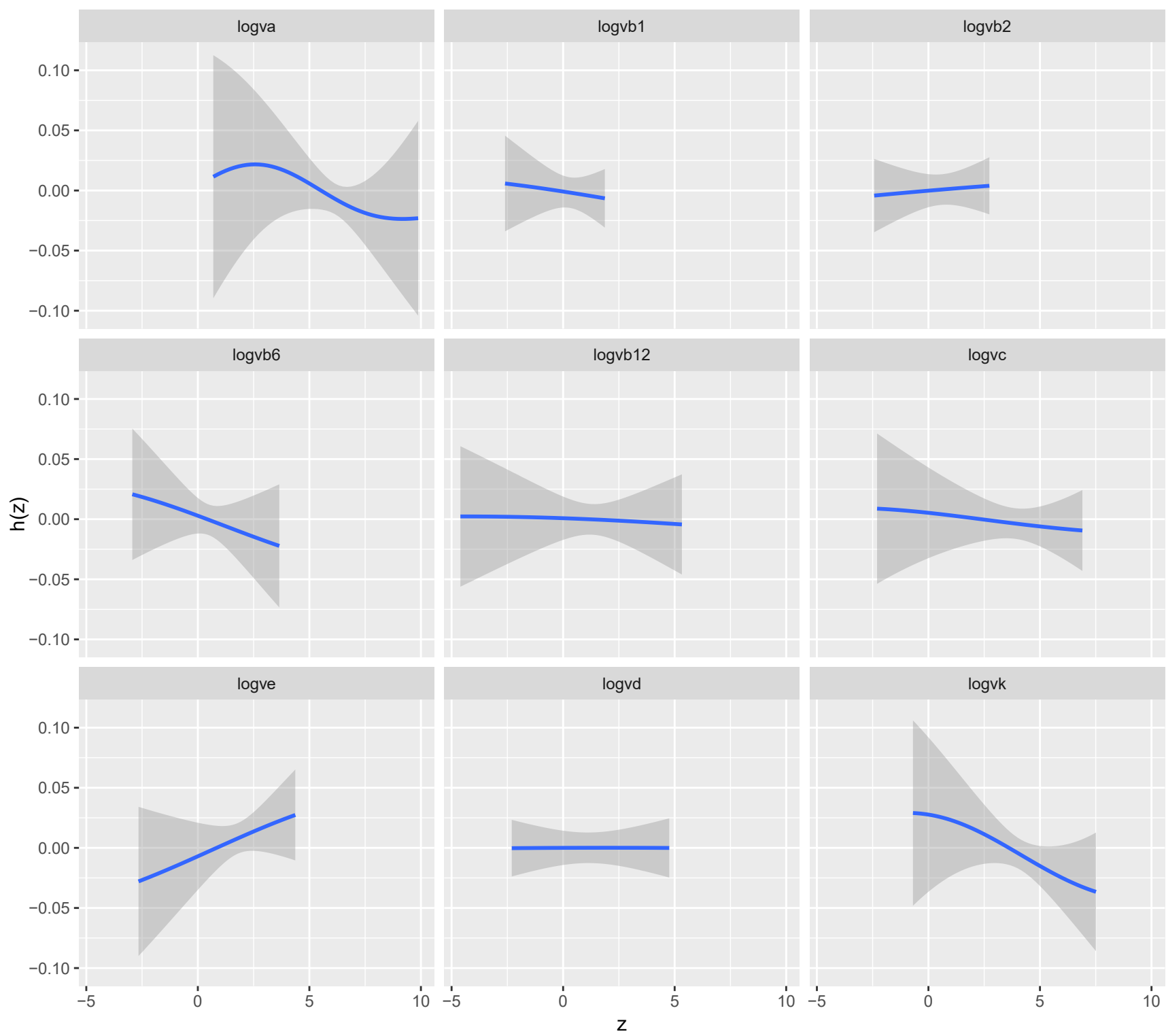

Supplement: Supplementary file 2 [file Image_2.pdf]

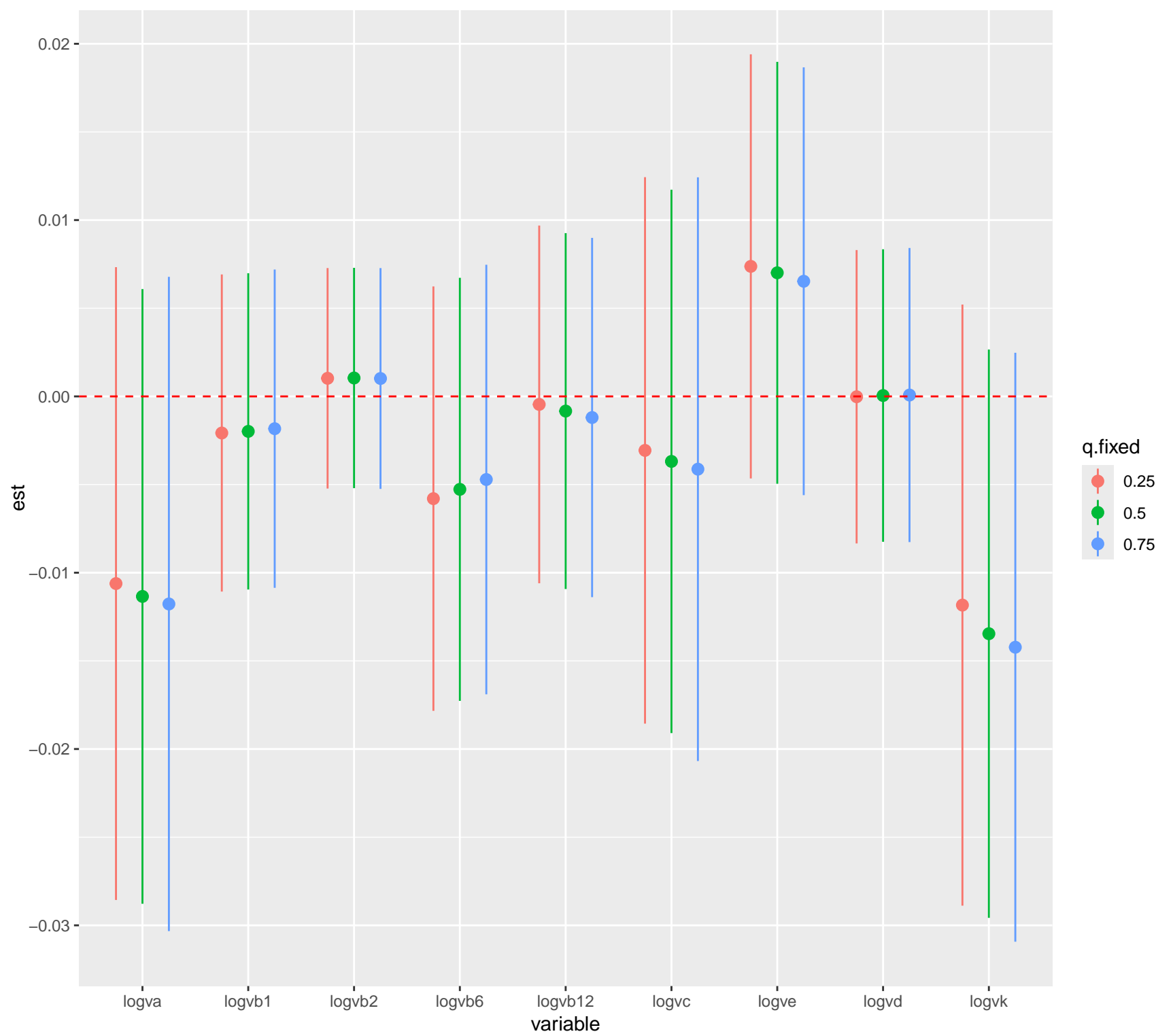

Supplement: Supplementary file 3 [file Image_3.pdf]

h(expos1 | quantiles of expos2)

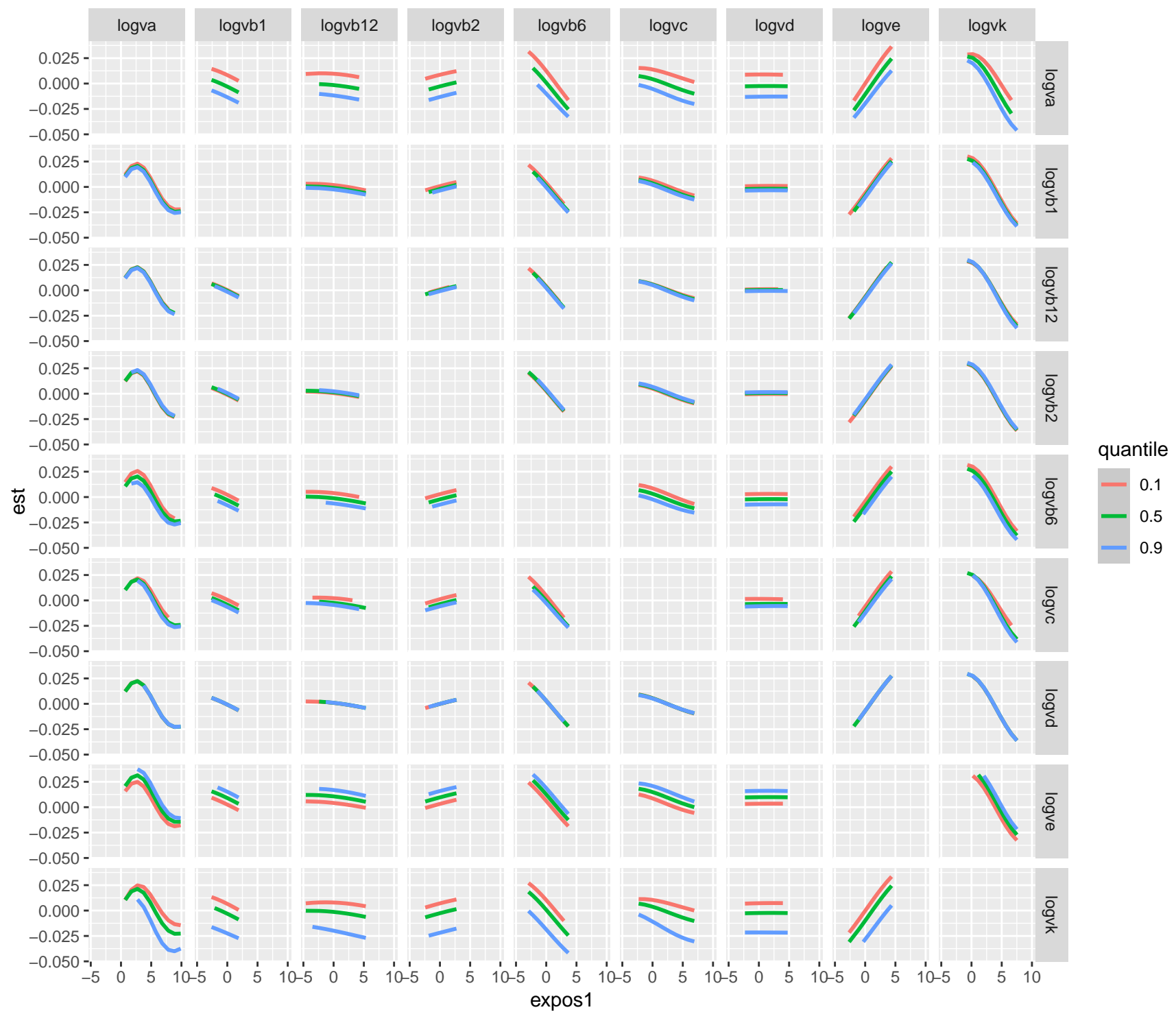

Supplement: Supplementary file 4 [file Image_4.pdf]

A

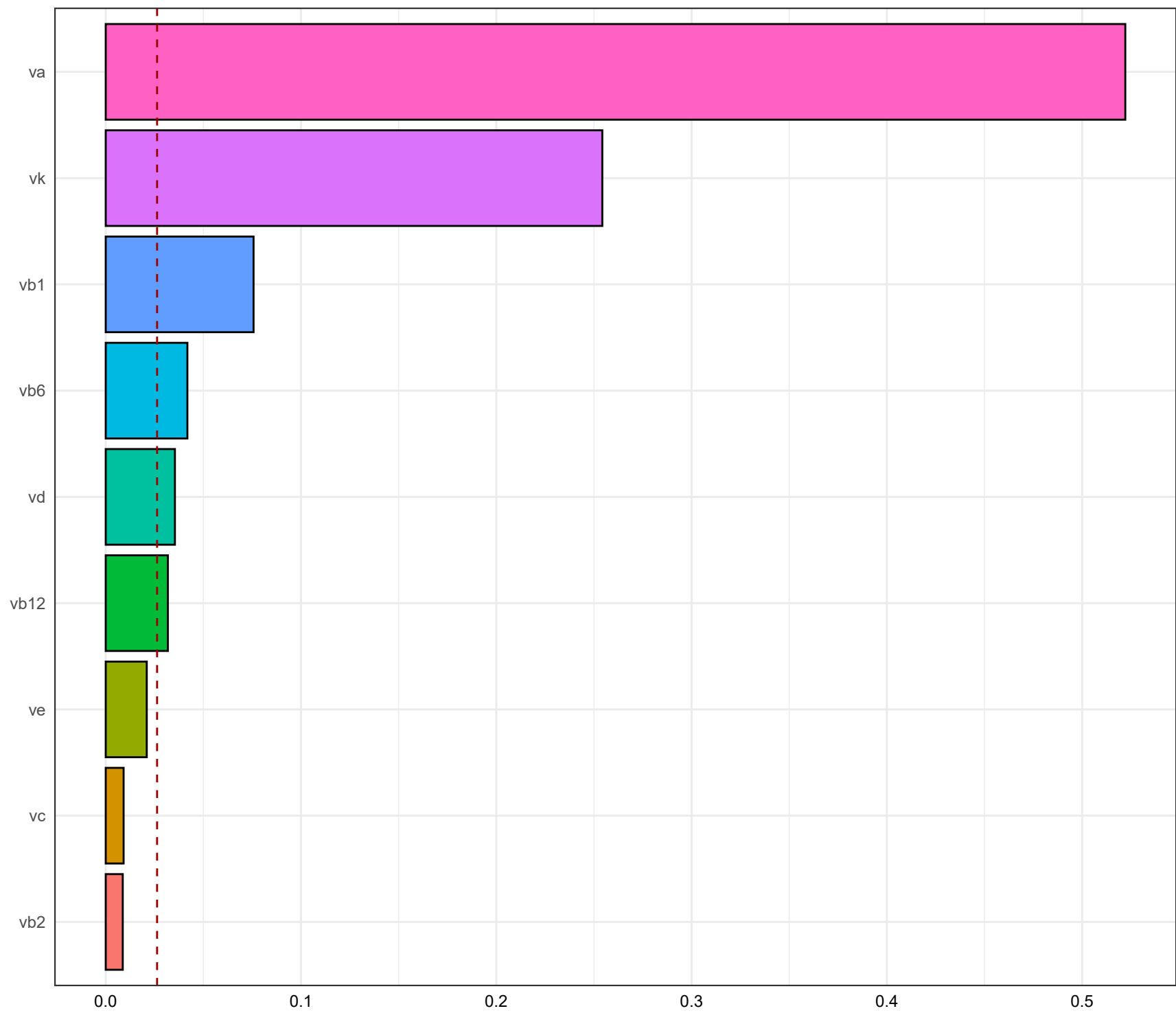

Supplement: Supplementary file 5 [file Image_5.pdf]
