# Supplementary material for: Associations between the intake of single and multiple dietary vitamins and depression risk among populations with chronic kidney disease
Source: Front Nutr. 2025 Feb 4;12:1492829. doi: 10.3389/fnut.2025.1492829 (PMC11832393; doi:10.3389/fnut.2025.1492829)
Supplement: Supplementary file 6 [file Table_1.DOCX]

**Table S1. The calculation of variance inflation factor (VIF)**

| Variables | VIF |
| --- | --- |
| Vitamin A | 3.6812941 |
| Vitamin B1 | 3.2268662 |
| Vitamin B2 | 4.3908439 |
| Vitamin B6 | 3.7181005 |
| Vitamin B12 | 3.0722422 |
| Vitamin C | 1.5440016 |
| Vitamin D | 3.2176123 |
| Vitamin E | 2.5932454 |
| Vitamin K | 3.1131444 |
| Age | 2.1544754 |
| Sex | 2.6230396 |
| Ethnicity | 2.3908001 |
| Marital status | 1.9206799 |
| Poverty-income ratio | 1.9016061 |
| Education level | 3.1894899 |
| Smoking status | 2.3914812 |
| Drinking status | 1.614936 |
| Diabetes | 1.6431127 |
| Hyperlipidemia | 1.7157254 |
| Hypertension | 3.3218004 |

**Table S2.** Baseline characteristics based on the CKD stages of eGFR.

| Variables | | Participants based on the staging of eGFR, No. (%) | | | | | | | | | | *P*-value | |
| --- | --- | --- | --- | --- | --- | --- | --- | --- | --- | --- | --- | --- | --- |
|  |  | Stages 1 | | Stages 2 | | Stages 3 | | Stages 4 | | Stages 5 | |  |  |
| Age, years | |  | |  | |  | |  | |  | | <0.0001 | |
| ≤65 | | 778(88.71) | | 323(45.62) | | 327(23.87) | | 22(20.00) | | 34(58.62) | |  | |
| >65 | | 99(11.29) | | 385(54.38) | | 1043(76.13) | | 88(80.00) | | 24(41.38) | |  | |
| Sex | |  | |  | |  | |  | |  | | <0.001 | |
| Male | | 390(44.47) | | 370(52.26) | | 678(49.49) | | 37(33.64) | | 32(55.17) | |  | |
| Female | | 487(55.53) | | 338(47.74) | | 692(50.51) | | 73(66.36) | | 26(44.83) | |  | |
| Ethnicity | |  | |  | |  | |  | |  | | <0.0001 | |
| White | | 289(32.95) | | 357(50.42) | | 880(64.23) | | 63(57.27) | | 15(25.86) | |  | |
| Mexican | | 177(20.18) | | 83(11.72) | | 87(6.35) | | 10(9.09) | | 7(12.07) | |  | |
| Black | | 244(27.82) | | 153(21.61) | | 246(17.96) | | 28(25.45) | | 29(50.00) | |  | |
| Other | | 167(19.04) | | 115(16.24) | | 157(11.46) | | 9(8.18) | | 7(12.07) | |  | |
| Marital status | |  | |  | |  | |  | |  | | 0.04 | |
| Married | | 412(46.98) | | 349(49.29) | | 722(52.70) | | 53(48.18) | | 23(39.66) | |  | |
| Other | | 465(53.02) | | 359(50.71) | | 648(47.30) | | 57(51.82) | | 35(60.34) | |  | |
| Education level | |  | |  | |  | |  | |  | | 0.53 | |
| Less than high school graduate | | 278(31.70) | | 226(31.92) | | 413(30.15) | | 44(40.00) | | 18(31.03) | |  | |
| High school graduate or general equivalency diploma | | 213(24.29) | | 169(23.87) | | 338(24.67) | | 29(26.36) | | 16(27.59) | |  | |
| Some college or above | | 386(44.01) | | 313(44.21) | | 619(45.18) | | 37(33.64) | | 24(41.38) | |  | |
| PIR | |  | |  | |  | |  | |  | | <0.0001 | |
| <1.0 | | 260(29.65) | | 175(24.72) | | 234(17.08) | | 23(20.91) | | 15(25.86) | |  | |
| ≥1.0 | | 617(70.35) | | 533(75.28) | | 1136(82.92) | | 87(79.09) | | 43(74.14) | |  | |
| Smoking status | |  | |  | |  | |  | |  | | <0.0001 | |
| Never | | 440(50.17) | | 340(48.02) | | 670(48.91) | | 50(45.45) | | 35(60.34) | |  | |
| Former | | 166(18.93) | | 242(34.18) | | 566(41.31) | | 49(44.55) | | 16(27.59) | |  | |
| Now | | 271(30.90) | | 126(17.80) | | 134(9.78) | | 11(10.00) | | 7(12.07) | |  | |
| Drinking status | |  | |  | |  | |  | |  | | <0.0001 | |
| Never | | 118(13.45) | | 121(17.09) | | 243(17.74) | | 35(31.82) | | 15(25.86) | |  | |
| Former | | 188(21.44) | | 216(30.51) | | 442(32.26) | | 42(38.18) | | 22(37.93) | |  | |
| Now | | 571(65.11) | | 371(52.40) | | 685(50.00) | | 33(30.00) | | 21(36.21) | |  | |
| Diabetes | |  | |  | |  | |  | |  | | <0.0001 | |
| No | | 555(63.28) | | 396(55.93) | | 822(60.00) | | 46(41.82) | | 21(36.21) | |  | |
| Yes | | 322(36.72) | | 312(44.07) | | 548(40.00) | | 64(58.18) | | 37(63.79) | |  | |
| Hyperlipidemia | |  | |  | |  | |  | |  | | <0.0001 | |
| No | | 215(24.52) | | 134(18.93) | | 183(13.36) | | 14(12.73) | | 16(27.59) | |  | |
| Yes | | 662(75.48) | | 574(81.07) | | 1187(86.64) | | 96(87.27) | | 42(72.41) | |  | |
| Hypertension | |  | |  | |  | |  | |  | | <0.0001 | |
| No | | 428(48.80) | | 156(22.03) | | 257(18.76) | | 12(10.91) | | 6(10.34) | |  | |
| Yes | | 449(51.20) | | 552(77.97) | | 1113(81.24) | | 98(89.09) | | 52(89.66) | |  | |
| Depression | |  | |  | |  | |  | |  | | 0.01 | |
| No | | 763(87.00) | | 620(87.57) | | 1245(90.88) | | 94(85.45) | | 48(82.76) | |  | |
| Yes | | 114(13.00) | | 88(12.43) | | 125(9.12) | | 16(14.55) | | 10(17.24) | |  | |
| Vitamin Types, Median (IQR) |  | |  | |  | |  | |  | |  | |  |
| Vitamin A, μg | | 413.00(471.00) | | 506.00(532.00) | | 485.50(494.25) | | 454.00(432.25) | | 332.00(446.50) | | <0.0001 | |
| Vitamin B1, mg | | 1.31(0.98) | | 1.35(0.93) | | 1.27(0.90) | | 1.16(0.85) | | 1.10(0.74) | | <0.01 | |
| Vitamin B2, mg | | 1.71(1.24) | | 1.74(1.09) | | 1.72(1.20) | | 1.48(0.94) | | 1.40(0.93) | | <0.0001 | |
| Vitamin B6, mg | | 1.67(1.29) | | 1.65(1.25) | | 1.55(1.16) | | 1.39(0.95) | | 1.14(0.91) | | <0.0001 | |
| Vitamin B12, μg | | 3.60(4.01) | | 3.79(4.12) | | 3.53(3.57) | | 2.95(3.27) | | 2.63(3.02) | | <0.01 | |
| Vitamin C, mg | | 46.60(85.30) | | 57.95(95.40) | | 50.05(80.57) | | 61.10(109.00) | | 32.35(57.10) | | <0.001 | |
| Vitamin D, μg | | 3.00(4.70) | | 3.40(4.60) | | 3.20(4.10) | | 2.70(4.18) | | 1.80(3.85) | | 0.03 | |
| Vitamin E, mg | | 6.05(5.90) | | 6.31(5.25) | | 5.76(5.00) | | 4.53(4.11) | | 4.90(3.16) | | <0.0001 | |
| Vitamin K, ug | | 52.50(76.70) | | 55.25(72.04) | | 57.05(77.40) | | 45.75(73.07) | | 45.95(52.72) | | 0.1 | |
| Total vitamin intake, mg | | 61.48(90.54) | | 63.54(84.91) | | 70.76(99.22) | | 71.61(108.62) | | 42.48(62.49) | | <0.001 | |

PIR, poverty-income ratio; IQR, interquartile range (75th quartile minus 25th quartile)

**Table S3 GroupPIP and CondPIP of nine dietary vitamins for BKMR model.**

| Variables | group | groupPIP | condPIP |
| --- | --- | --- | --- |
| Vit A | 1 | 0.64 | 1.00 |
| Vit B1 | 2 | 0.53 | 0.22 |
| Vit B2 | 2 | 0.53 | 0.14 |
| Vit B6 | 2 | 0.53 | 0.42 |
| Vit B12 | 2 | 0.53 | 0.17 |
| Vit C | 3 | 0.29 | 1.00 |
| Vit E | 4 | 0.61 | 0.30 |
| Vit D | 2 | 0.53 | 0.06 |
| VitK | 4 | 0.61 | 0.70 |

**Table S4 mean weight of nine dietary vitamins for WQS model.**

| Variables | mean weight |
| --- | --- |
| Vit A | 0.522 |
| Vit B1 | 0.076 |
| Vit B2 | 0.009 |
| Vit B6 | 0.042 |
| Vit B12 | 0.032 |
| Vit C | 0.009 |
| Vit D | 0.035 |
| Vit E | 0.021 |
| Vit K | 0.254 |
